# Development of a risk prediction model for acute kidney injury in liver transplant recipients

**DOI:** 10.3389/fsurg.2025.1683424

**Published:** 2025-11-18

**Authors:** Dahui Wang, Xin Chen, Yanchen Tang, Wei Xiao, Kangqing Xu, Jiehua Deng

**Affiliations:** 1 Department of Plastic and Aesthetic Surgery, The Second Affiliated Hospital of Guilin Medical University, Guilin, Guangxi Zhuang Autonomous Region, China; 2 Department of Anesthesiology, The First Affiliated Hospital, Sun Yat-sen University, Guangzhou, Guangdong, China; 3 Neurobiology of Pain Research Group, Institute of Neurosciences, School of Medicine, University of Barcelona, Barcelona, Spain; 4 Department of Anesthesiology, The Second Affiliated Hospital of Hainan Medical University, Haikou, China; 5 Department of Anesthesiology, The Affiliated Changde Hospital, Hengyang Medical School, University of South China, Hengyang, China

**Keywords:** liver transplantation, acute kidney injury, risk factors, nomogram, predictive model

## Abstract

**Objective:**

To develop and internally validate a nomogram for early postoperative prediction of acute kidney injury (AKI) within 7 days after orthotopic liver transplantation (LT).

**Methods:**

We retrospectively analyzed 500 orthotopic liver transplants at the First Affiliated Hospital of Sun Yat-sen University (January 1, 2016–April 30, 2022). Patients were randomly split into training (*n* = 352) and validation (*n* = 148) cohorts for same-center internal validation using a random-split design. AKI within 7 postoperative days was defined by KDIGO serum-creatinine criteria only (KDIGO-SCr) because urine-output data were incomplete. Candidate predictors were screened using least absolute shrinkage and selection operator (LASSO) and entered into multivariable logistic regression to build a parsimonious nomogram for early postoperative (first 6–12 h) risk stratification and monitoring. Performance was assessed by AUC and calibration; decision-curve analysis illustrated relative net benefit without prespecified thresholds or actions.

**Results:**

BMI, operation time, intraoperative urine volume, and postoperative levels of urea nitrogen, blood ammonia, and procalcitonin were identified as independent risk factors for AKI after LT (*P* < 0.05). The nomogram demonstrated good discrimination, calibration, and clinical usefulness in both the training and validation cohorts, with an AUC of 0.769 (95% CI: 0.715–0.823) in the training cohort and 0.704 (95% CI: 0.618–0.790) in the validation cohort.

**Conclusion:**

The nomogram predictive model developed in this study shows good accuracy and can be conveniently applied for early identification and risk prediction of acute kidney injury following liver transplantation.

## Introduction

Liver transplantation is the most effective treatment for patients with advanced liver failure, significantly improving overall survival rates and quality of life (1). However, liver transplantation can lead to various complications. One complication is the postoperative acute kidney injury which has many researches done on this topic (2). A meta-analysis in 2019 reported that in recent years, the incidence rates of AKI post-liver transplantation and severe AKI requiring RRT were 40.8% and 7% (3), respectively. AKI after liver transplantation is closely associated with delayed renal function recovery, increased transplantation failures, extended mechanical ventilation time, prolonged postoperative ICU stay, increased 30-day postoperative mortality, and decreased long-term survival rates of patients (4–6).

Preoperative, intraoperative, and postoperative factors all contribute to the occurrence of AKI after transplantation. Preoperative factors include patient's own conditions. Pre-existing conditions such as viral hepatitis, hepatic encephalopathy, cerebrovascular disease, overweight, and diabetes increase the risk of AKI (7–9). The duration of the surgery (10) and the surgical technique used also influence the occurrence of AKI. Some studies indicate that the piggyback technique significantly reduces the likelihood of acute renal failure after liver transplantation (11, 12). Postoperative factors include postoperative hypotension, postoperative infections, among others (13, 14).

Currently, there are many research on novel biological markers related to renal function and kidney injury such as NGAL, KIM-1, and Cystatin C (15). However, for ESLD patients, there's a lack of biochemical indicators with high sensitivity, strong specificity, and broad clinical applicability for renal function assessment. Therefore, early identification and diagnosis of postoperative AKI and immediate therapeutic measures can be crucial to improve patient prognosis (16). Therefore, to aid clinicians in early identification and risk stratification during the perioperative period, and enable effective intervention targeting various high-risk factors, it is essential to develop an accurate model for predicting the occurrence of AKI. This study aims to investigate the risk factors of OLT-AKI through a retrospective analysis of the clinical data of 500 OLT patients, based on the KDIGO diagnostic criteria. The objective of the prediction model was to achieve early individualized assessment of the risk of developing OLT-AKI.

## Patients and methods

### Patients selection

**Figure 1:**
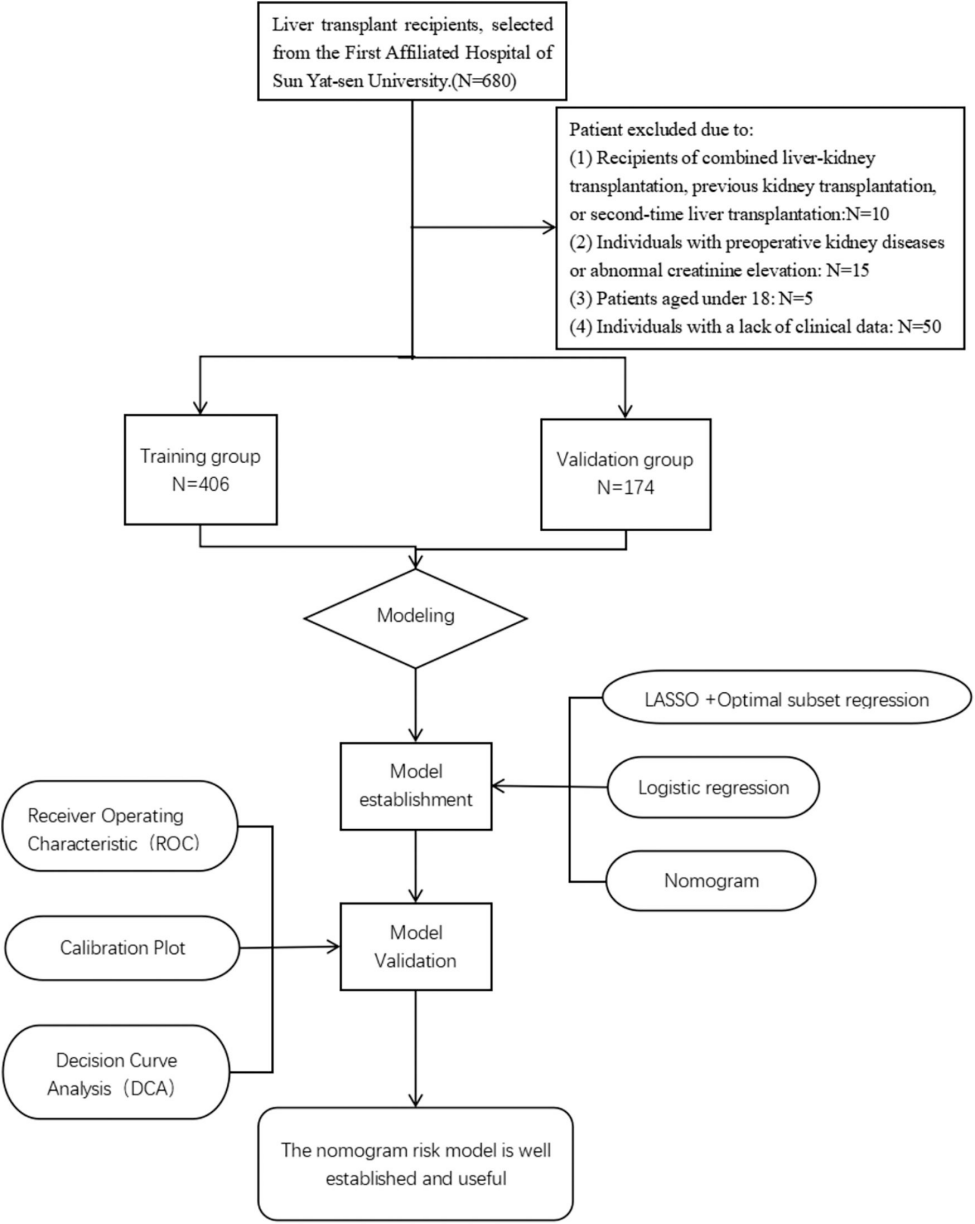
Flow diagram of study design.

Clinical data of patients undergoing their first liver transplantation at the First Affiliated Hospital of Sun Yat-sen University from January 1, 2016 to April 30, 2022 was collected. Post-liver transplantation AKI was diagnosed based on the diagnostic criteria established by the Kidney Disease: Improving Global Outcomes (KDIGO) in 2012. Exclusion criteria: (1) recipients of combined liver-kidney transplantation, previous kidney transplantation, or second-time liver transplantation; (2) preoperative kidney diseases or abnormal creatinine elevation; (3) patients aged under 18; (4) lack of clinical data. A total of 500 patients were included in this study. Donor characteristics such as age, sex, and graft quality were recorded when available. However, information regarding extended-criteria donor (ECD) status and graft steatosis was not uniformly documented in the database and was therefore not analyzed.Candidates were divided into a training group (*n* = 352) and a validation group (*n* = 148). The research flow chart is shown in Figure 1. Based on the C-statistic of 0.8, an estimated 10 risk factors were projected. According to previous literature, the incidence rate of AKI is 36.1% (17), resulting in an estimated sample size of 355. Due to the limitations of retrospective studies which prevent accurate collection of urine volume for each patient, this study only used Scr as the diagnostic marker for AKI which still aligns to the KDIGO diagnostic standards. This study was approved by the Medical Ethics Committee of the First Affiliated Hospital of Sun Yat-sen University (Approval Number: 2022-468).

### Data collection

Preoperative data included age, gender, body mass index (BMI), ASA classification, primary disease and comorbidities, preoperative medication, and laboratory tests. Intraoperative data encompassed the surgical method, duration of low central venous pressure, duration of hypotension, duration of surgery and anesthesia, intraoperative medication, volume of various fluids administered, total intraoperative blood loss, and total urine output. “Inferior vena cava (IVC) time” was defined as the duration of temporary occlusion of the recipient's inferior vena cava during the anhepatic phase of liver transplantation. Postoperative data covered postoperative AST and ALT peaks, coagulation parameters, and infection indicators. Prognostic data included postoperative hepatic insufficiency, incidence of hepatic encephalopathy, ICU stay duration, duration of postoperative mechanical ventilation, reintubation rate, secondary laparotomy, postoperative CRRT requirement, and mortality.

### Definition and outcome

The surgical approach utilized either the classic orthotopic liver transplantation (OLT) or the modified piggyback OLT. In the classic OLT, the recipient's retrohepatic inferior vena cava (IVC) was completely removed and replaced with the donor IVC, requiring temporary suprahepatic and infrahepatic caval clamping. In contrast, the modified piggyback OLT preserved the recipient's IVC by anastomosing the donor hepatic vein cuff to the sidewall of the recipient IVC, thereby maintaining partial venous return and avoiding total caval interruption. All procedures were performed by the same experienced surgical team.

AKI was diagnosed using KDIGO criteria based on serum creatinine changes within 7 days post-transplantation. Because urine output data were not consistently available, the KDIGO urine output criterion was not applied; thus, intraoperative urine volume was analyzed as a perioperative variable rather than part of the AKI definition.MELD, ALBI, and CTP scores were utilized to evaluate end-stage liver disease (18). The ALBI (Albumin–Bilirubin) score is an objective indicator reflecting liver synthetic and excretory function, calculated from serum albumin and total bilirubin, and is considered a simple and reproducible index for grading liver function without subjective parameters (19). The Child–Turcotte–Pugh (CTP) score, which incorporates serum bilirubin, albumin, INR, ascites, and encephalopathy, has been widely used to assess hepatic reserve and predict postoperative outcomes (20). In addition to the MELD score, which mainly reflects liver disease severity and short-term mortality risk, the inclusion of ALBI and CTP scores provides a more comprehensive assessment of hepatic functional reserve, thereby improving the accuracy of preoperative evaluation (21). MELD = 3.8 × Ln(TBIL) + 9.6 × Ln(SCr) + 11.2 × Ln(INR) + 6.4 × etiology (0 for alcoholic and cholestatic liver disease, 1 for others), ALBI = (log10 bilirubin × 0.66) + (albumin × −0.085), CTP = 0.957 × loge(total bilirubin) + 0.378 × loge(albumin) + 1.12 × loge(PT-INR) + 0.643 (1 point if ascites present, otherwise 0) + 0.871 (graded from 0–3 based on encephalopathy level). However, the diagnostic criteria for early postoperative liver dysfunction (EAD) lacks a unified standard. According to Olthoff et al. (22), EAD diagnosis is determined by the following: (1) Total bilirubin (TBIL) ≥ 10 mg/dl on postoperative day 7; (2) International Normalized Ratio (INR) ≥ 1.6 on postoperative day 7; (3) Peak aspartate aminotransferase (AST) or peak alanine aminotransferase (ALT) > 2,000 U/L within 7 days post-surgery. Any of the above criteria confirms an EAD diagnosis.

### Statistical analysis

All statistical analyses were performed using Stata/SE 17.0 (College Station, TX, USA) and R version 4.2.2 software (https://www.r-project.org/). Continuous variables were expressed as mean ± standard deviation (SD) and compared using Student's *t* test when approximately normally distributed, as assessed by the Shapiro–Wilk test. Non-normally distributed variables were presented as median [interquartile range (IQR, P25–P75)] and compared using the Mann–Whitney *U* test. Categorical variables were expressed as counts (percentages) and compared using the *χ*² test or Fisher's exact test. Differences were considered statistically significant at *P* < 0.05. The use of mean ± SD for continuous variables followed previous liver transplantation studies and facilitated comparability across cohorts with approximately normal data distributions.

The LASSO regression model allowed for the risk factors (non-zero coefficients) to be obtained from the clinical data characteristics. The penalty term was determined by 10-fold cross-validation, selecting the penalty that yielded the smallest mean square error (“glmnet” package). The main tuning parameter was set as follows: type. Measure (loss to use for cross-validation) = “default”, family = “binomial”, nfolds = 10. The optimal model, with the fewest variables, was identified based on *λ*1 se as the criterion. The multifactorial logistic regression method was then used for further screening of independent risk factors. A nomogram was also established by the final model through the “rms” package of R software. The area under the curve (AUC) of the receiver operating characteristic curve (ROC) alongside calibration curves and decision curves were used to internally and externally validate the nomogram prediction model to judge its' discriminative ability, calibration, and clinical usefulness.In addition to the AUC, the model's sensitivity, specificity, positive predictive value (PPV), and negative predictive value (NPV) were also calculated to further evaluate its discriminative performance.

## Results

### Patient baseline characteristics and prognosis

From January 1, 2016 to April 30, 2022, our hospital had a total of 500 patients undergoing LT. Based on the inclusion criteria, a total of 500 patients were added into the analysis, with 352 in the training group and 148 in the validation group. The detailed flowchart is shown in Figure 1. The overall characteristics of the two groups of patients are summarized in Table 1. In the training and validation cohorts, postoperative AKI occurred in 117 patients (33.2%) and 50 patients (33.8%) respectively. For analysis of postoperative characteristics and outcomes, patients were divided into two groups: AKI group and non-AKI group (Table 2). Compared to the non-AKI group, patients in the AKI group were more likely to undergo dialysis, had higher chances of reintubation, a higher mortality rate, prolonged hospital stay, prolonged postoperative ICU stay, longer duration of mechanical ventilation after surgery, and prolonged postoperative hospitalization. The likelihood of postoperative liver dysfunction and the incidence of hepatic encephalopathy had also increased. (*P* < 0.05).

**Table 1:** Clinical characteristics of the study population in the training and validation groups.

Term	level	Overall	testdata	triandata	*p*
*n*		500	148	352	
AKI (%)	0	333 (66.6)	98 (66.2)	235 (66.8)	0.906
	1	167 (33.4)	50 (33.8)	117 (33.2)	
Gender (%)	1	448 (89.6)	132 (89.2)	316 (89.8)	0.845
	2	52 (10.4)	16 (10.8)	36 (10.2)	
Age [mean (SD)]		52.06 (10.87)	52.03 (11.32)	52.07 (10.69)	0.967
BMI [mean (SD)]		23.21 (3.56)	23.40 (3.54)	23.13 (3.57)	0.453
ASAgrade (%)	2	46 (9.2)	13 (8.8)	33 (9.4)	0.513
	3	311 (62.2)	90 (60.8)	221 (62.8)	
	4	139 (27.8)	45 (30.4)	94 (26.7)	
	5	4 (0.8)	0 (0.0)	4 (1.1)	
MELDscore [mean (SD)]		24.26 (7.73)	24.45 (8.33)	24.19 (7.47)	0.727
ALBIscore [mean (SD)]		−2.05 (0.61)	−2.06 (0.63)	−2.04 (0.60)	0.779
CTPscore [mean (SD)]		7.44 (2.31)	7.55 (2.64)	7.39 (2.16)	0.501
abdominal.surgery (%)	0	345 (69.0)	101 (68.2)	244 (69.3)	0.812
	1	155 (31.0)	47 (31.8)	108 (30.7)	
hypertension (%)	0	429 (85.8)	125 (84.5)	304 (86.4)	0.578
	1	71 (14.2)	23 (15.5)	48 (13.6)	
CAD (%)	0	485 (97.0)	143 (96.6)	342 (97.2)	0.748
	1	15 (3.0)	5 (3.4)	10 (2.8)	
CVD (%)	0	494 (98.8)	145 (98.0)	349 (99.1)	0.271
	1	6 (1.2)	3 (2.0)	3 (0.9)	
hyperlipidemia (%)	0	497 (99.4)	148 (100.0)	349 (99.1)	0.26
	1	3 (0.6)	0 (0.0)	3 (0.9)	
diabetes (%)	0	430 (86.0)	128 (86.5)	302 (85.8)	0.839
	1	70 (14.0)	20 (13.5)	50 (14.2)	
Hepatitis.B (%)	0	77 (15.4)	20 (13.5)	57 (16.2)	0.449
	1	423 (84.6)	128 (86.5)	295 (83.8)	
viral.cirrhosis (%)	0	140 (28.0)	39 (26.4)	101 (28.7)	0.594
	1	360 (72.0)	109 (73.6)	251 (71.3)	
preLiverF (%)	0	445 (89.0)	131 (88.5)	314 (89.2)	0.822
	1	55 (11.0)	17 (11.5)	38 (10.8)	
cholangioc.arcinoma (%)	0	490 (98.0)	145 (98.0)	345 (98.0)	0.978
	1	10 (2.0)	3 (2.0)	7 (2.0)	
Liver.cancer (%)	0	169 (33.8)	48 (32.4)	121 (34.4)	0.675
	1	331 (66.2)	100 (67.6)	231 (65.6)	
Ascites (%)	0	291 (58.2)	82 (55.4)	209 (59.4)	0.411
	1	209 (41.8)	66 (44.6)	143 (40.6)	
preHE (%)	0	484 (96.8)	142 (95.9)	342 (97.2)	0.482
	1	16 (3.2)	6 (4.1)	10 (2.8)	
UGIB (%)	0	448 (89.6)	131 (88.5)	317 (90.1)	0.606
	1	52 (10.4)	17 (11.5)	35 (9.9)	
PVT (%)	0	449 (89.8)	134 (90.5)	315 (89.5)	0.723
	1	51 (10.2)	14 (9.5)	37 (10.5)	
infect (%)	0	263 (52.6)	73 (49.3)	190 (54.0)	0.342
	1	237 (47.4)	75 (50.7)	162 (46.0)	
cholelithiasis (%)	0	329 (65.8)	96 (64.9)	233 (66.2)	0.775
	1	171 (34.2)	52 (35.1)	119 (33.8)	
Hepatitis.C (%)	0	484 (96.8)	146 (98.6)	338 (96.0)	0.128
	1	16 (3.2)	2 (1.4)	14 (4.0)	
alcoholic.cirrhosis (%)	0	482 (96.4)	145 (98.0)	337 (95.7)	0.221
	1	18 (3.6)	3 (2.0)	15 (4.3)	
preBblock (%)	0	445 (89.0)	133 (89.9)	312 (88.6)	0.689
	1	55 (11.0)	15 (10.1)	40 (11.4)	
pre.hormone (%)	0	386 (77.2)	116 (78.4)	270 (76.7)	0.684
	1	114 (22.8)	32 (21.6)	82 (23.3)	
Pre.Diuretic (%)	0	262 (52.4)	74 (50.0)	188 (53.4)	0.486
	1	238 (47.6)	74 (50.0)	164 (46.6)	
Hb [mean (SD)]		114.55 (29.19)	113.66 (31.00)	114.93 (28.44)	0.657
RBCcount [mean (SD)]		3.79 (1.04)	3.83 (1.14)	3.77 (0.99)	0.542
WBC [mean (SD)]		5.22 (2.91)	5.25 (3.02)	5.20 (2.87)	0.876
PLT [mean (SD)]		117.33 (89.46)	122.53 (96.67)	115.15 (86.29)	0.4
BUN [mean (SD)]		5.37 (3.35)	5.29 (3.24)	5.40 (3.40)	0.732
Scr [mean (SD)]		73.43 (24.29)	76.95 (28.79)	71.95 (22.02)	0.036
pre.UA [mean (SD)]		321.71 (125.61)	317.47 (135.79)	323.49 (121.24)	0.625
pre.ALB [mean (SD)]		36.88 (5.48)	36.97 (4.91)	36.85 (5.71)	0.825
pre.TBIL [mean (SD)]		113.22 (188.32)	118.75 (196.13)	110.90 (185.18)	0.671
pre.ALT [mean (SD)]		55.86 (123.27)	47.80 (46.81)	59.24 (143.68)	0.344
pre.AST [mean (SD)]		79.92 (180.99)	69.02 (59.20)	84.51 (212.20)	0.383
pre.GGT [mean (SD)]		123.11 (156.63)	118.24 (140.56)	125.16 (163.05)	0.652
pre.LDH [mean (SD)]		271.19 (524.42)	228.85 (81.92)	288.99 (622.17)	0.242
pre.ALP [mean (SD)]		147.04 (130.66)	140.47 (111.11)	149.80 (138.11)	0.466
pre.CHE [mean (SD)]		4,061.63 (2,084.22)	4,231.47 (2,224.11)	3,990.23 (2,021.55)	0.238
pre.ammonia [mean (SD)]		47.24 (29.63)	48.63 (32.06)	46.65 (28.58)	0.495
pre.PT [mean (SD)]		16.41 (6.45)	16.84 (8.27)	16.22 (5.52)	0.326
pre.PT. [mean (SD)]		65.50 (24.00)	65.21 (25.23)	65.62 (23.50)	0.861
pre.INR [mean (SD)]		1.42 (0.61)	1.46 (0.78)	1.40 (0.52)	0.371
pre.APTT [mean (SD)]		41.79 (18.20)	42.57 (18.70)	41.47 (18.00)	0.534
pre.TT [mean (SD)]		19.48 (2.59)	19.76 (3.00)	19.37 (2.39)	0.119
pre.FIB [mean (SD)]		2.31 (1.30)	2.22 (1.26)	2.34 (1.32)	0.358
Surgery.time [mean (SD)]		449.91 (104.45)	448.41 (113.70)	450.54 (100.48)	0.836
tranexamic.acid (%)	0	236 (47.2)	61 (41.2)	175 (49.7)	0.082
	1	264 (52.8)	87 (58.8)	177 (50.3)	
sevoflurane (%)	0	26 (5.2)	6 (4.1)	20 (5.7)	0.454
	1	474 (94.8)	142 (95.9)	332 (94.3)	
Dexmedetomidine (%)	0	208 (41.6)	60 (40.5)	148 (42.0)	0.755
	1	292 (58.4)	88 (59.5)	204 (58.0)	
Intra.Diuretic (%)	0	190 (38.0)	59 (39.9)	131 (37.2)	0.577
	1	310 (62.0)	89 (60.1)	221 (62.8)	
dopamine (%)	0	250 (50.0)	76 (51.4)	174 (49.4)	0.695
	1	250 (50.0)	72 (48.6)	178 (50.6)	
norepinephrine (%)	0	15 (3.0)	3 (2.0)	12 (3.4)	0.408
	1	485 (97.0)	145 (98.0)	340 (96.6)	
adrenaline (%)	0	256 (51.2)	73 (49.3)	183 (52.0)	0.586
	1	244 (48.8)	75 (50.7)	169 (48.0)	
SBPtime [mean (SD)]		38.24 (41.06)	35.07 (35.20)	39.58 (43.27)	0.262
MAPtime [mean (SD)]		82.40 (79.11)	81.27 (79.17)	82.87 (79.19)	0.836
hepatic.portal.occlusion.time [mean (SD)]		55.01 (18.51)	53.72 (20.39)	55.56 (17.66)	0.31
Inferior.vena.cava.time [mean (SD)]		49.58 (15.87)	48.60 (17.50)	49.99 (15.14)	0.37
bleeding [mean (SD)]		1,914.89 (1,911.72)	1,773.48 (1,867.82)	1,974.35 (1,929.40)	0.284
crystalloid [mean (SD)]		4,813.45 (1,982.42)	4,711.66 (1,665.62)	4,856.25 (2,102.14)	0.457
colloid (%)	0	194 (38.8)	62 (41.9)	132 (37.5)	0.358
	1	306 (61.2)	86 (58.1)	220 (62.5)	
trans.ALB [mean (SD)]		343.48 (176.75)	330.26 (172.43)	349.03 (178.48)	0.279
trans.RBC [mean (SD)]		1,178.86 (1,494.13)	1,184.84 (1,998.04)	1,176.35 (1,225.46)	0.954
trans.FFP [mean (SD)]		1,509.50 (912.85)	1,551.35 (996.20)	1,491.90 (876.35)	0.507
trans.cryoprecipitate [mean (SD)]		5.73 (7.84)	4.98 (7.58)	6.05 (7.94)	0.162
trans.PLT [mean (SD)]		0.79 (2.45)	0.89 (2.38)	0.74 (2.47)	0.556
trans.h.kg [mean (SD)]		20.39 (4.40)	20.13 (3.85)	20.50 (4.61)	0.394
LCVP.time [mean (SD)]		103.87 (107.51)	90.68 (95.80)	109.41 (111.74)	0.075
Classic.in.situ (%)		376 (75.2)	114 (77.0)	262 (74.4)	0.540
Improved.camel.back(%)		124 (24.8)	34 (23.0)	90 (25.6)	
total.urine [mean (SD)]		1,620.36 (885.14)	1,628.65 (816.57)	1,616.88 (913.52)	0.892
post.PCT [mean (SD)]		17.36 (28.37)	16.71 (23.67)	17.63 (30.15)	0.740
post.BUN [mean (SD)]		6.82 (3.23)	6.87 (3.68)	6.81 (3.03)	0.849
post.Ammonia [mean (SD)]		37.61 (26.43)	37.61 (29.86)	37.61 (24.90)	0.999

**Table 2:** Postoperative outcomes of the patients in AKI and non-AKI groups.

Term	level	Overall	Non-AKI	AKI	*p*
*n*		500	333	167	<0.001
Post-liver dysfunction (%)	0	284 (56.8)	210 (63.1)	74 (44.3)	
	1	216 (43.2)	123 (36.9)	93 (55.7)	0.015
Post-hepatic encephalopathy(%)	0	446 (89.2)	305 (91.6)	141 (84.4)	
	1	54 (10.8)	28 (8.4)	26 (15.6)	<0.001
In hospital day [mean (SD)]		37.98 (20.05)	35.74 (18.62)	42.46 (22.02)	<0.001
POD [mean (SD)]		26.02 (13.01)	24.35 (11.59)	29.37 (14.96)	<0.001
ICU time [mean (SD)]		66.04 (92.06)	44.18 (47.23)	109.62 (134.70)	<0.001
Ventilation time [mean (SD)]		37.19 (76.12)	20.78 (27.32)	69.92 (119.62)	<0.001
RE intubation (%)	0	479 (95.8)	330 (99.1)	149 (89.2)	
	1	21 (4.2)	3 (0.9)	18 (10.8)	<0.001
Dialysis (%)	0	470 (94.0)	333 (100.0)	137 (82.0)	
	1	30 (6.0)	0 (0.0)	30 (18.0)	0.001
Second laparotomy (%)	0	487 (97.4)	330 (99.1)	157 (94.0)	
	1	13 (2.6)	3 (0.9)	10 (6.0)	<0.001
Hospital death (%)	0	488 (97.6)	332 (99.7)	156 (93.4)	
	1	12 (2.4)	1 (0.3)	11 (6.6)	0.989
Amount of ascites [mean (SD)]		168.90 (730.94)	169.22 (666.81)	168.26 (846.62)	<0.001

### Selection of variables for AKI post LT

**Figure 2:**
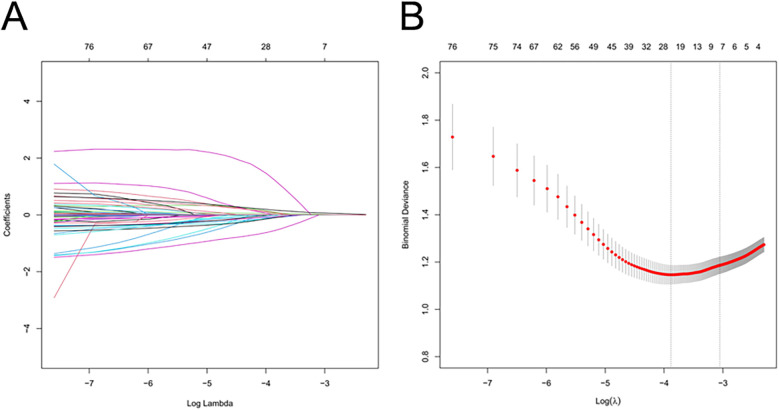
Feature selection of LT patients using the LASSO logistic regression model. **(A)** A Lasso coefficient profile plot was built for the prediction of AKI after LT. **(B)** The optimal parameter (λ) was selected by the LASSO model using 10-fold cross-validation via 1 standard error of the minimum criteria.

Using the LASSO algorithm, 6 potential risk factors were selected from 80 variables, with the chosen Lambda.1se being 0.0201 (Figures 2A,B). After multivariate logistic regression analysis, the 6 factors remained independently associated with the risk of AKI in LT patients (Table 3). Although variables such as hepatic portal occlusion time, inferior vena cava time, MELD score, and pre-FIB were statistically significant in the univariate analysis, they lost significance after LASSO selection and multivariable logistic regression due to collinearity with other predictors (e.g., operation time, postoperative BUN, and PCT) or overlapping explanatory power. Preoperative variables (BMI), intraoperative variables (surgical time, total urine output during surgery), and postoperative variables (postoperative PCT, postoperative BUN, postoperative blood ammonia) were identified to be independent risk factors for AKI after LT.

**Table 3:** Multivariable logistic regression analysis of predicting AKI after LT in the training cohort.

Term	Crude OR (95% CI)	uni-*P* value	Adj OR (95% CI)	multi-*P* value
Preoperative variables				
Abdominal surgery1	0.651 (0.391, 1.064)	0.092		
Age	1.006 (0.985, 1.027)	0.591		
Gender2	1.005 (0.469, 2.051)	0.99		
BMI	1.072 (1.007, 1.142)	0.03	1.114 (1.004, 1.239)	0.043
ASAgrade3	0.616 (0.293, 1.320)	0.204		
ASAgrade4	0.668 (0.296, 1.522)	0.331		
ASAgrade5	4.071 (0.465, 87.250)	0.245		
ALBI score	1.457 (1.003, 2.130)	0.049	0.692 (0.390, 1.233)	0.21
MELD score	1.034 (1.004, 1.065)	0.025	0.982 (0.908, 1.061)	0.65
CTP score	1.081 (0.976, 1.198)	0.132		
Alcoholic cirrhosis1	1.004 (0.307, 2.898)	0.994		
Viral cirrhosis1	1.104 (0.677, 1.824)	0.694		
Hepatitis.B1	0.756 (0.423, 1.374)	0.349		
Hepatitis.C1	1.534 (0.494, 4.516)	0.439		
Liver.cancer1	0.765 (0.482, 1.217)	0.255		
Cholangioc arcinoma1	0.329 (0.017, 1.957)	0.306		
cholelithiasis1	0.969 (0.603, 1.544)	0.895		
UGIB1	0.912 (0.415, 1.892)	0.811		
CAD1 (Coronary artery disease)	1.351 (0.340, 4.824)	0.646		
CVD1(Cerebrovascular disease)	1.004 (0.046, 10.589)	0.997		
diabetes1	1.554 (0.835, 2.854)	0.158		
hypertension1	1.242 (0.651, 2.317)	0.501		
hyperlipidemia1	4.070 (0.386, 88.106)	0.254		
preHE1(Hepatic encephalopathy)	2.054 (0.561, 7.523)	0.263		
preLiverF1(Liver failure)	2.204 (1.113, 4.369)	0.023	1.742 (0.540, 5.727)	0.353
PVT1(Portal vein thrombosis)	0.616 (0.266, 1.305)	0.227		
Ascites1	1.081 (0.687, 1.694)	0.735		
Pre Diuretic1	1.080 (0.692, 1.684)	0.736		
Pre hormone1(preoperative hormone use)	0.982 (0.575, 1.649)	0.945		
preBblock1(beta-blocker)	1.393 (0.697, 2.718)	0.337		
Pre ALB	0.984 (0.946, 1.023)	0.413		
Pre ALT	1.000 (0.999, 1.002)	0.639		
Pre AST	1.000 (0.998, 1.001)	0.869		
Pre ALP	1.000 (0.998, 1.001)	0.857		
Pre CHE	1.000 (1.000, 1.000)	0.71		
Pre FIB	0.829 (0.686, 0.989)	0.044	1.096 (0.838, 1.420)	0.493
Pre GGT	1.000 (0.998, 1.001)	0.896		
Pre LDH	1.000 (1.000, 1.001)	0.413		
Pre INR	1.448 (0.956, 2.199)	0.079		
pre.PT	1.035 (0.996, 1.077)	0.081		
pre.PT.	0.993 (0.983, 1.002)	0.146		
pre.TT	1.103 (1.007, 1.210)	0.035	1.054 (0.903, 1.229)	0.504
Pre APTT	1.009 (0.997, 1.022)	0.128		
pre.UA	1.000 (0.998, 1.001)	0.629		
Pre ammonia	0.996 (0.988, 1.004)	0.333		
Pre TBIL	1.001 (1.000, 1.002)	0.031	1.000 (0.997, 1.003)	0.991
Hb(hemoglobin)	0.995 (0.987, 1.003)	0.21		
PLT (platelet count)	0.998 (0.995, 1.000)	0.097		
WBC	1.020 (0.944, 1.101)	0.603		
RBC count	0.805 (0.637, 1.010)	0.064		
Pre-Scr	0.994 (0.983, 1.005)	0.294		
Pre-BUN	1.009 (0.943, 1.075)	0.787		
infect1	0.957 (0.612, 1.493)	0.848		
Intraoperative Variables				
Surgery time	1.004 (1.002, 1.007)	<0.001	1.004 (1.000, 1.008)	0.029
Classic *in situ* or improved camel back2(surgical technique)	1.075 (0.644, 1.774)	0.778		
Hepatic portal occlusion time	1.014 (1.002, 1.027)	0.024	1.009 (0.986, 1.032)	0.44
Inferior vena cava time	1.016 (1.002, 1.032)	0.027	0.996 (0.970, 1.023)	0.776
LCVP time (Low central venous pressure time)	0.999 (0.997, 1.001)	0.37		
MAP time (Mean arterial pressure time)	1.002 (0.999, 1.004)	0.242		
SBP time (Systolic BP time)	1.000 (0.995, 1.005)	0.979		
adrenaline1	0.850 (0.543, 1.325)	0.473	adrenaline1	0.850 (0.543, 1.325)
dopamine1	1.156 (0.742, 1.806)	0.521		
norepinephrine1	0.996 (0.307, 3.794)	0.994		
Dexmedetomidine1	0.819 (0.524, 1.284)	0.383		
sevoflurane1	1.527 (0.575, 4.793)	0.424		
Tranexamic acid1	0.958 (0.614, 1.494)	0.851		
Intra Diuretic1(intraoperative diuretic)	1.216 (0.768, 1.944)	0.407		
colloid1	1.554 (0.975, 2.507)	0.067		
crystalloid	1.000 (1.000, 1.000)	0.034	1.000 (1.000, 1.000)	0.88
Bleeding (blood loss)	1.000 (1.000, 1.000)	<0.001	1.000 (1.000, 1.000)	0.271
Total urine (urine output)	1.000 (0.999, 1.000)	0.012	1.000 (0.999, 1.000)	0.018
Trans RBC	1.000 (1.000, 1.000)	0.001	1.000 (1.000, 1.000)	0.654
Trans PLT	1.026 (0.936, 1.120)	0.555		
Trans FFP	1.000 (1.000, 1.001)	0.001	1.000 (1.000, 1.001)	0.234
Trans cryoprecipitate	1.028 (1.000, 1.056)	0.051		
Trans ALB(if postoperative infusion)	1.000 (0.999, 1.002)	0.438		
trans.h.kg (intraoperative fluid volume/body weight ratio)	0.948 (0.899, 0.997)	0.043	1.026 (0.941, 1.117)	0.551
Postoperative Variables				
Post BUN	1.203 (1.105, 1.323)	<0.001	1.179 (1.071, 1.317)	0.002
post.PCT	1.019 (1.010, 1.029)	<0.001	1.017 (1.008, 1.028)	0.001
Post Ammonia	1.022 (1.012, 1.032)	<0.001	1.015 (1.003, 1.027)	0.014

### Construction of nomogram model for predicting AKI in patients

**Figure 3:**
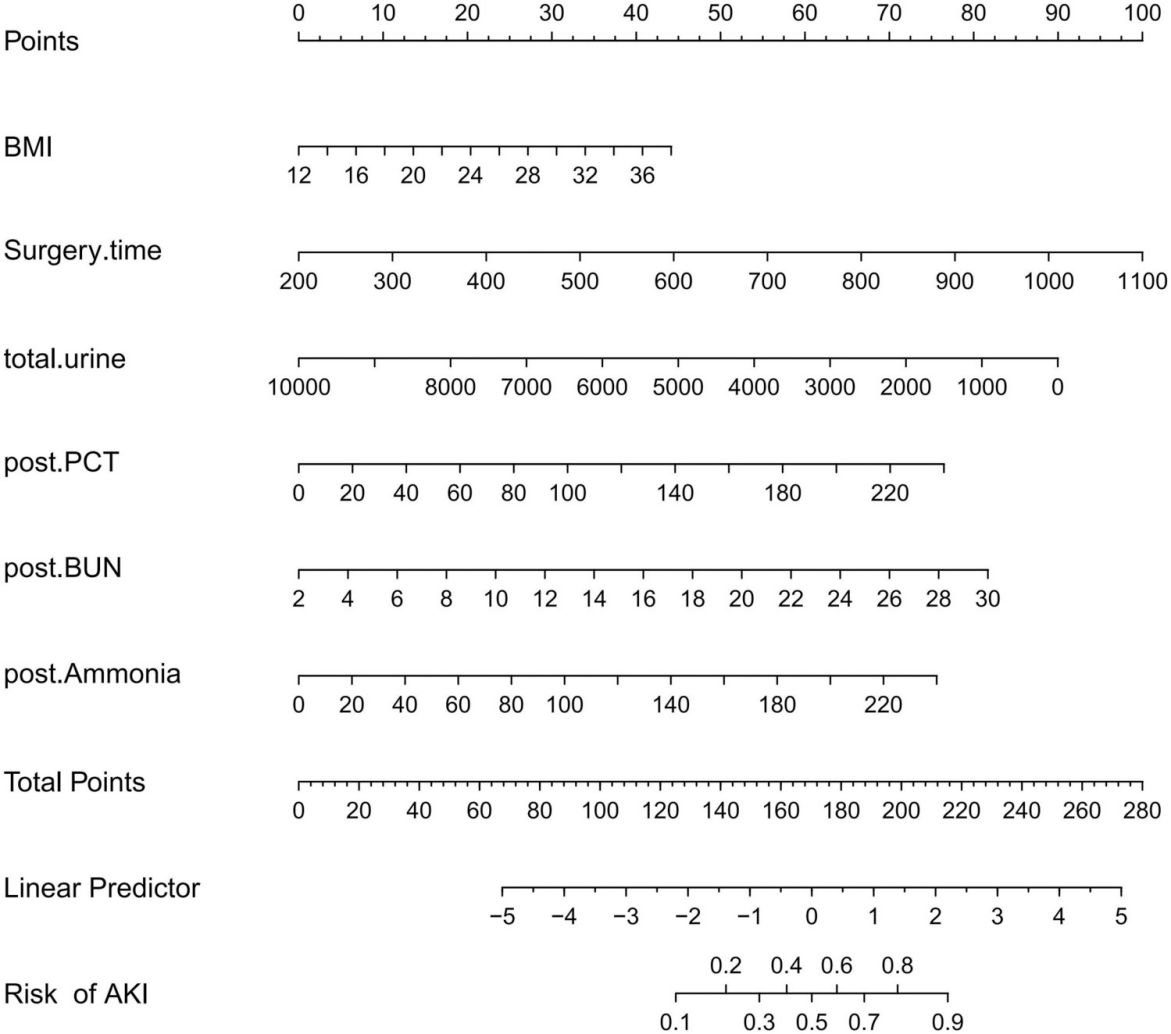
A risk factors of BMI, surgery time, total urine, postoperative PCT, postoperative BUN, postoperative ammonia for nomogram prediction model.

Taking the development of AKI as the dependent variable, the variables determined in the multivariate logistic regression analysis were used as predictive variables, and the total scores of each factor in the nomogram predicting the risk of AKI were calculated. The risk of AKI is represented by the total score, with a scoring range from 0–280. The scores of each risk factor are shown in Figure 3. By combining the scores of BMI, surgery duration, total urine volume, post-operative PCT, post-operative BUN, and post-operative blood ammonia, the corresponding AKI risk can be determined.

### Performance and validation of the AKI nomogram model post-LT

**Figure 4:**
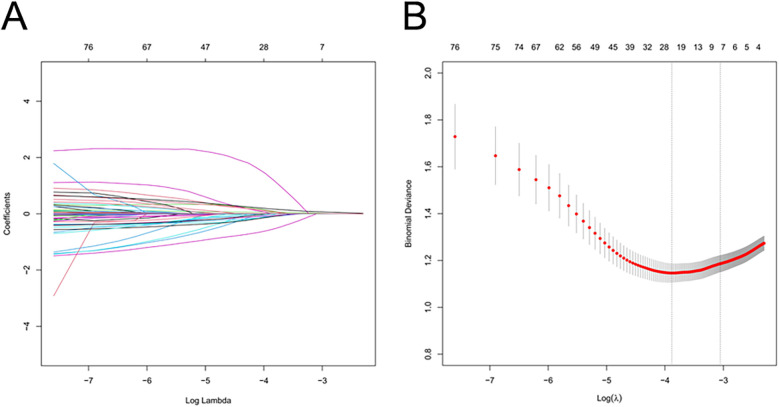
Receiver operating characteristic (ROC) curve validates the nomogram prediction for the risk of acute kidney injury post liver transplantation. The blue curve **(A)** represents the performance of the nomogram in the training group, while the red curve **(B)** demonstrates its performance in the validation group.

**Figure 5:**
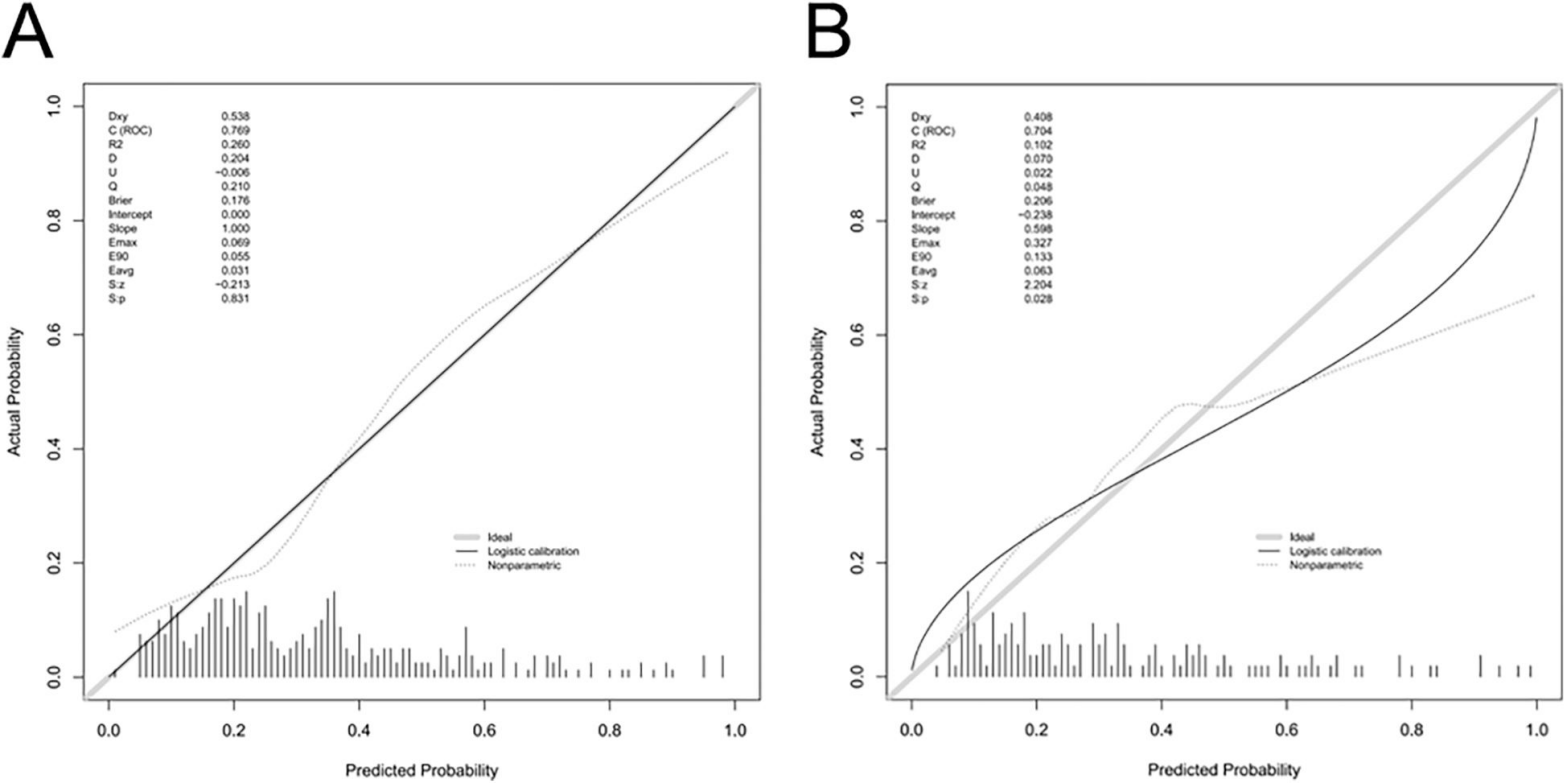
Calibration curves of the predictive nomogram for AKI risk. The *y*-axis represents the actual diagnosed cases of AKI, and the *x*-axis represents the predicted risk of AKI. The diagonal dotted line denotes a perfect prediction by an ideal model. The solid lines represent the performance of the nomogram in the training group **(A)** and validation group **(B)**, with a closer alignment to the diagonal dotted line indicating a more accurate prediction. I am creating a legend title; please assist me with the translation.

The AUC of the ROC analysis for the training group and validation group were 0.769 (95% CI: 0.715–0.823) and 0.704 (95% CI: 0.618–0.790), respectively (Figures 4A,B) indicating that the model demonstrated excellent accuracy in estimating the probability of AKI following LT. Additionally, the sensitivity, specificity, PPV, and NPV of the model were calculated to complement the AUC results, all of which demonstrated consistent and acceptable predictive performance (see Supplementary Table S1). The calibration curve of the nomogram is presented in Figures 5A,B, and revealing good agreement between the predicted and observed outcomes.

### Clinical application of the AKI nomogram model post-LT

**Figure 6:**
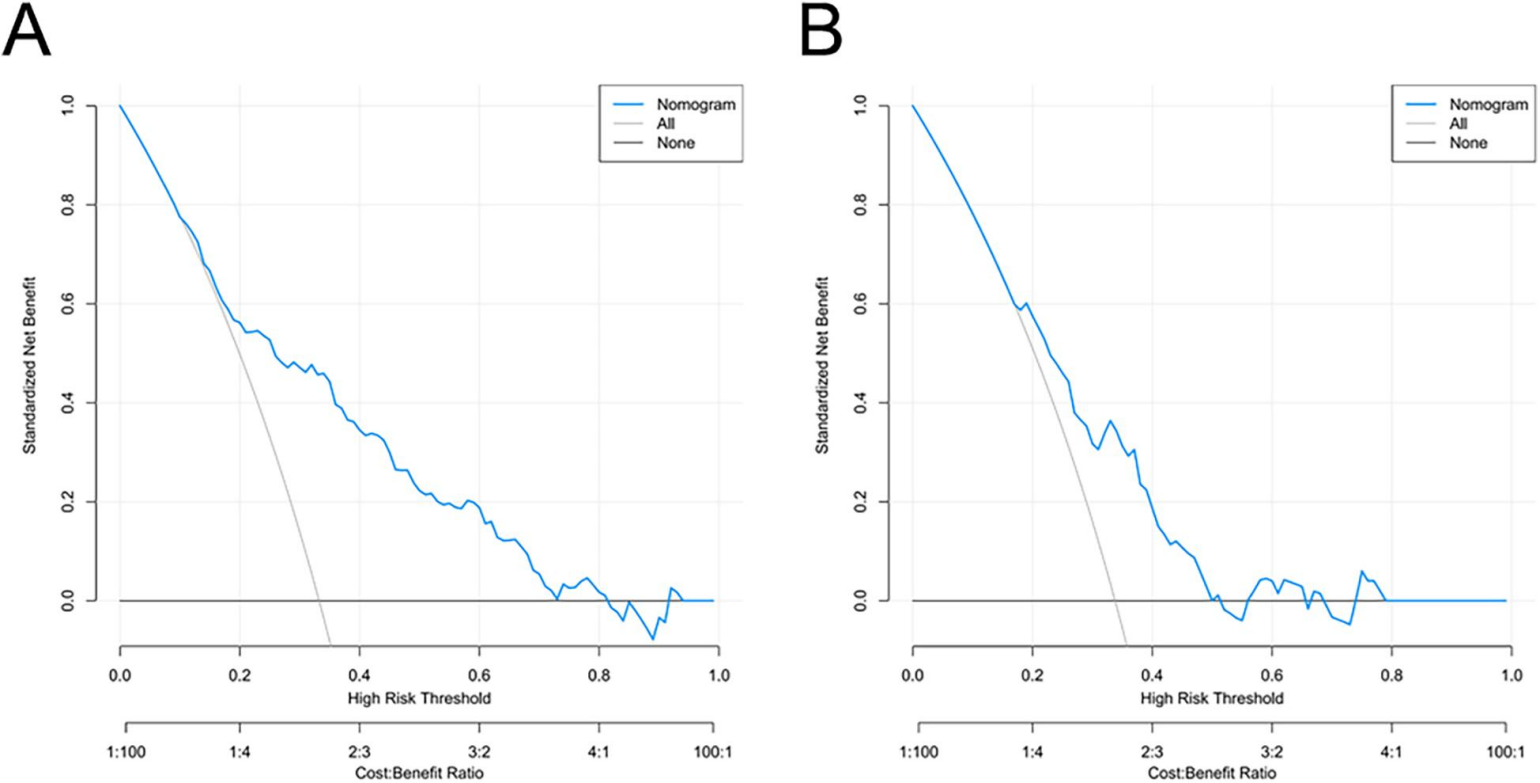
Decision curve analysis for the AKI risk nomogram. The *y*-axis measures the net benefit. The thick solid line represents the assumption that all patients do not have AKI, the thin solid line represents the assumption that all patients have AKI, and the blue line represents the risk nomogram. **(A)** originates from the training group, and **(B)** originates from the validation group.

Decision curve analysis (DCA) was used to evaluate the clinical usefulness of the nomogram by quantifying the net benefit across a range of threshold probabilities. When the threshold probability values were within 10%–82% in the training cohort and 18%–50% in the validation cohort, the nomogram provided a higher net benefit compared with the “treat-all” or “treat-none” strategies, indicating good potential for assisting individualized clinical decision-making in predicting postoperative AKI risk (Figures 6A,B).

## Discussion

OLT is the ultimate effective treatment for various categories of liver failure which include acute, sub-acute, or chronic liver failure. In recent years, there has been an upward trend in liver transplantation (23). However, various short-term and long-term complications after LT remain to be critical issues and they needed to be addressed in current clinical practice. AKI is one of the common complications after LT surgery (24). In this study, the independent risk factors for OLT-AKI include: BMI, surgical duration, total urine volume during surgery, postoperative PCT, postoperative BUN, and postoperative blood ammonia. Based on the results of the Logistic regression, a risk prediction model for OLT-AKI was constructed and a nomogram was plotted. The results indicate that the prediction model has good calibration and discrimination capabilities, allowing risk stratification and earlier identification. It further allows the clinicians to implement effective interventions improving the patients' prognosis.

In this study, the incidence of AKI after liver transplantation was 32.4%. The morbidity rate previously reported by Zhu and others was 61.1%. The higher AKI incidence reported in other clinical studies may be due to the adoption of the newly released KDIGO criteria. Recent reports on AKI after liver transplantation have uniformly adopted this criterion with incidence rates ranging from 12.3%–56.6% (2, 3) which is consistent with the findings of this study. Meanwhile, this study indicates that early AKI post-transplantation not only increases in-hospital mortality, prolongs discharge time, and postoperative mechanical ventilation duration, but it is also closely associated with adverse outcomes such as secondary laparotomy and re-intubation which is again, consistent with the previous research findings.

BMI, identified as the sole preoperative independent risk factor in this study, has been previously reported (9). High BMI predisposes individuals to cardiovascular diseases, hypertension, and diabetes (25). Overweight patients, particularly those undergoing liver transplantation, are prone to postoperative complications (26). However, some studies have shown that obesity is associated with a higher long-term postoperative survival rate. Underweight patients significantly suffer from worse postoperative complications and higher mortality rates (27). Several epidemiological studies suggest that obesity provides a protective effect for critically ill patients. In this context, Sleeman et al. observed in an established extracorporeal circulation model of pigs that obese pigs showed reduced kidney functions and tissue apoptosis after surgery. The animal models and clinical studies have yielded contradictory findings to each other (28). Our findings revealed that individuals with a high BMI undergoing orthotopic liver transplant are at a higher risk of postoperative AKI. This susceptibility may be attributed to the association between obesity, heightened oxidative stress, elevated proinflammatory cytokine levels, and potential endothelial dysfunction, all of which could contribute to the development of AKI (29, 30). From a clinical perspective, patients who are obese or overweight can pose challenges in the surgical setting, leading to increased complexity of the procedure, potentially prolonging the operation time, and raising the risk of postoperative complications. Moreover, fluid resuscitation in obese patients presents a challenge due to the uncertainty surrounding whether fluid infusion should be adjusted based on actual weight or formula weight, and this ambiguity can impact renal perfusion (9).

In our research, we identified two intraoperative variables as risk factors: operation time and the total urine output during surgery. In this model, operation time played the most significant role in predicting postoperative AKI. Operation time was determined by various factors, such as the difficulty of the operation, the technical proficiency of the operator, the pathophysiology of the patient, and the management by the anesthesiologist during the perioperative period. This might indirectly result in varying threshold values obtained in previous studies when operation time is considered as an independent risk factor (10, 31). Although operative time was identified as an independent risk factor in our multivariable analysis, it may act as a surrogate marker for intraoperative physiological stress or surgical complexity rather than a direct cause of postoperative AKI. Factors such as massive blood loss, intraoperative hemorrhagic shock, ischemia–reperfusion injury, and the use or dose of vasopressors could partially explain the observed association between longer surgery duration and renal dysfunction (32, 33). Furthermore, urine output is a macroscopic representation of renal perfusion, and intraoperative urine output is influenced by various factors such as blood volume and hemodynamics. Previous studies (10) have shown that intraoperative urine output of <2 ml/(kg·h) is an independent risk factor for postoperative AKI in liver transplant patients.

Previous studies have used the Framingham Risk-scheme to develop a risk prediction model for AKI occurrence after deceased donor LT. However, this study did not include relevant indicators after LT (34). In our model, three post-operative risk factors were established as standalone predictive indicators which include PCT after surgery, BUN, and blood ammonia. PCT is a regular diagnostic measure for sepsis due to its' excellent specificity and sensitivity, and PCT is also closely associated with sepsis (35). It is a marker of inflammation with high sensitivity and precision which aids in detecting infectious diseases earlier, assessing inflammation, evaluating disease prognosis, and guiding accurate drug use. A recent meta-analysis on PCT labeled it as a potential predictive factor for AKI (36). For conditions like traumatic brain injury, septic shock, acute pancreatitis, primary percutaneous coronary interventions post-coronavirus infection, and acute Type A aortic dissection, PCT holds substantial predictive value for AKI (37–39, 42). However, the forecasting capability of PCT for post-liver transplant AKI hasn't been discussed. In our research, PCT demonstrates potential predictive capacity for AKI following liver transplantation, which might be associated with its low molecular weight. Under normal circumstances, the kidneys can efficiently remove (PCT) from the bloodstream. However, when kidney function is compromised, the clearance rate of PCT from the plasma decreases, resulting in the accumulation of this substance in the bloodstream. Since this topic hasn't been extensively explored in previous research, it is imperative to collect more cases and even consider conducting multicenter collaborative studies to corroborate the association between PCT and AKI. Furthermore, determining the ideal threshold for predicting post-liver transplant AKI introduces a novel perspective and avenue for investigation.

Postoperative blood ammonia levels are significantly correlated with the occurrence of AKI after liver transplantation. Research has found that high blood ammonia can damage the kidneys through multiple mechanisms. For instance, elevated blood ammonia during chronic liver disease can directly interact with renal glomerular cells, leading to glomerular damage (40), and ammonia also plays a crucial role on renal tubulointerstitial fibrosis. Other studies have demonstrated that hyperammonemia can facilitate the progression of kidney injury through the activation of the complement cascade and the stimulating impact of ammonia on kidney growth (41). Yoon Sook Lee (42) et al.'s research suggested that preoperative blood ammonia can serve as a predictor for AKI after liver transplantation, which contradicted the findings from our study. These discrepancies may arise from factors such as sample size in the research and the timing of preoperative blood ammonia collection. Hence, additional research may be necessary to investigate the reasons behind the discrepancies with previous study findings and to delve more deeply into the relationship between blood ammonia levels and AKI following liver transplantation under various clinical scenarios.

Previous models predicting post-operative AKI often included preoperative urea nitrogen, but there is limited research on whether postoperative urea can also be a predictor. A previous study from Portugal reported that postoperative blood urea nitrogen can be a strong predictor for acute kidney injury after pediatric cardiac surgery (43). Lu Haiyang et al. found that after liver transplantation, the AUC analysis showed that BUN had a good distinction for Stage 3 AKI. However, there were significant differences in the preoperative urea values between the AKI group and non-AKI group, which might lead to biased results (44). In our study, a significant correlation between elevated postoperative urea levels and postoperative AKI was observed, further supporting the importance of closely monitoring renal function in patients after liver transplantation, especially those with elevated postoperative urea levels. Timely renal-protective measures, including optimized fluid management and appropriate pharmacologic interventions, may help reduce the risk of postoperative AKI and improve recovery outcomes.

Furthermore, some predictors identified in this study—such as operation time and intraoperative urine volume—are potentially modifiable. Future studies should investigate whether optimizing these perioperative factors can causally reduce AKI risk, possibly through advanced causal inference frameworks such as target trial emulation (45).

The potential heterogeneity of the study population should also be acknowledged. Variations in baseline characteristics, perioperative management, and comorbidities among liver transplant recipients may influence the development of postoperative AKI. Although strict inclusion criteria and internal validation were applied, unmeasured heterogeneity might still have affected the observed associations. Future studies should perform subgroup analyses stratified by demographic or clinical factors (e.g., age, BMI, MELD score, or liver disease etiology) to verify the model's consistency across populations (46).

This study has several limitations. First, serum creatinine was used to define AKI, which may underestimate renal injury due to perioperative hemodilution or fluid shifts. Because urine-output data were unavailable, AKI was defined by KDIGO-SCr rather than full KDIGO; SCr-only ascertainment may misclassify AKI in post-LT high-fluid states and affect observed associations, so incidence estimates and predictor effects should be interpreted cautiously. We did not quantify the impact of excluding urine output; future work should externally validate the model in cohorts with complete urine-output capture and, where feasible, compare SCr-only with full KDIGO ascertainment. Second, this was a single-center retrospective analysis, so causal inference cannot be fully established. Third, detailed donor information such as extended-criteria donor (ECD) status and graft steatosis was not consistently available in the dataset, which may have introduced minor residual confounding (47). Fourth, the decision-curve analysis (DCA) serves only to visualize relative net benefit; no implementation inferences are made because decision thresholds and linked clinical actions were not prespecified. For future translation, thresholds should be prospectively defined with clinical stakeholders and explicitly mapped to actions relevant to transplant care (e.g., increased monitoring frequency and nephrotoxin avoidance at lower thresholds; early nephrology consultation; CRRT readiness; ICU resource prioritization at higher thresholds) and then evaluated in independent cohorts to confirm utility. Additionally, in line with TRIPOD, we report measurement timing, candidate-predictor rationale, modeling choices, and the full model specification with coefficients/intercept to enable independent use; calibration is shown graphically, and numerical calibration slope/intercept and the Brier score are not reported here and represent a reporting limitation to be addressed—together with optimism-corrected performance—in future temporal/multicenter external validation. Finally, although the nomogram showed good internal validation performance, external validation using independent cohorts was not available; thus, potential overfitting and limited generalizability cannot be excluded. Future multicenter studies are warranted to confirm the robustness of this model.

In summary, our study suggests a strong link between AKI and higher mortality rates as well as adverse outcomes. After liver transplantation, the acute kidney injury can be attributed to several independent risk factors such as BMI, surgery duration, total urine volume, post-surgery PCT, post-surgery BUN, and post-surgery blood ammonia. These individual determinants are incorporated into a nomogram, allowing for personalized risk assessment of acute kidney injury following orthotopic liver transplantation. Overall, this nomogram demonstrated good discrimination and calibration performance, as reflected by the AUC and calibration plots.

## Data Availability

The raw data supporting the conclusions of this article will be made available by the authors, without undue reservation.
