# Supplementary material for: Development of a risk prediction model for acute kidney injury in liver transplant recipients
Source: Front Surg. 2025 Nov 18;12:1683424. doi: 10.3389/fsurg.2025.1683424 (PMC12669099; doi:10.3389/fsurg.2025.1683424)
Supplement: Supplementary file 1 [file Supplementaryfile1.docx]

## Supplementary Table S1.

Sensitivity, specificity, positive predictive value (PPV), and negative predictive value (NPV) of the nomogram model for predicting postoperative acute kidney injury (AKI) after liver transplantation.

| Cohort | Sensitivity | Specificity | PPV | NPV |
| --- | --- | --- | --- | --- |
| Training | 0.78 | 0.71 | 0.69 | 0.79 |
| Validation | 0.73 | 0.68 | 0.67 | 0.75 |

Abbreviations: PPV, positive predictive value; NPV, negative predictive value; AKI, acute kidney injury.
